# *Codium fragile* Ameliorates High-Fat Diet-Induced Metabolism by Modulating the Gut Microbiota in Mice

**DOI:** 10.3390/nu12061848

**Published:** 2020-06-21

**Authors:** Jungman Kim, Jae Ho Choi, Taehwan Oh, Byungjae Ahn, Tatsuya Unno

**Affiliations:** 1 Faculty of Biotechnology, School of Life Sciences, SARI, Jeju National University, Jeju 63243, Korea; kjm5364@gmail.com; 2 Subtropical/Tropical Organism Gene Bank, Jeju National University, Jeju 63243, Korea; jaehochoi78@gmail.com; 3 Marine Biotechnology Research Center, Jeonnam Bioindustry Foundation, Wando 59108, Korea; sosoth2@hanmail.net (T.O.); chemeditech@hanmail.net (B.A.)

**Keywords:** *Codium fragile*, gut microbiota, high-fat diet, intestinal metabolism, obesity

## Abstract

*Codium fragile* (CF) is a functional seaweed food that has been used for its health effects, including immunostimulatory, anti-inflammatory, anti-obesity and anti-cancer activities, but the effect of CF extracts on obesity via regulation of intestinal microflora is still unknown. This study investigated anti-obesity effects of CF extracts on gut microbiota of diet-induced obese mice. C57BL/6 mice fed a high-fat (HF) diet were given CF extracts intragastrically for 12 weeks. CF extracts significantly decreased animal body weight and the size of adipocytes, while reducing serum levels of cholesterol and glucose. In addition, CF extracts significantly shifted the gut microbiota of mice by increasing the abundance of Bacteroidetes and decreasing the abundance of Verrucomicrobia species, in which the portion of beneficial bacteria (i.e., Ruminococcaceae, Lachnospiraceae and *Acetatifactor*) were increased. This resulted in shifting predicted intestinal metabolic pathways involved in regulating adipocytes (i.e., mevalonate metabolism), energy harvest (i.e., pyruvate fermentation and glycolysis), appetite (i.e., chorismate biosynthesis) and metabolic disorders (i.e., isoprene biosynthesis, urea metabolism, and peptidoglycan biosynthesis). In conclusion, our study showed that CF extracts ameliorate intestinal metabolism in HF-induced obese mice by modulating the gut microbiota.

## 1. Introduction

Obesity is caused by abnormal or excessive fat accumulation due to an imbalance between energy intake and energy consumption [1]. Obesity is known as a risk factor for the causes of diseases including metabolic syndrome, type II diabetes, hypercholesterolemia, and vascular diseases, which can lead to complications in several organs [2,3]. The development of obesity is closely linked to lifestyle and dietary habits. In particular, the energy imbalance between caloric intake and expenditure is the underlying cause of overweight and obesity, and increased consumption of high-density foods with high fat and sugar content and reduced physical activity promotes the prevalence of obesity worldwide [4].

Although limiting calories and increasing exercise are often effective interventions to combat obesity, they are not as effective for some because of physical inability or lack of willpower. Various functional foods or nutritional ingredients from both traditional and novel resources have been extensively studied to prevent and alleviate obesity without compromising the enjoyment of eating with large amounts of calories. So far, various methods have been implemented to manage overweight or obesity, but increasing attention has recently been paid to the roles of gut microbiota for treating obesity. Obesity is generally related with changes in the total composition of gut microbiota, and it has been found that obese people have less microbial diversity than lean people [5,6]. The precise mechanism is still unclear, but a correlation has been found between specific microbial communities and a development of obesity. A recent study has reported the evidence that gut microbes behave similarly to the endocrine organs and also act as important regulators of obesity by affecting the absorption of nutrients in the gut [7]. Furthermore, it has been reported that excessive fat intake contributes to a high amount of fat in the bloodstream, resulting in increased fat tissue and fat accumulation in the body, leading to lipid metabolic disorders and obesity [8]. Diet is critically involved in the regulation of the gut microbiota. The incidence of obesity and related disorders have been linked to alterations of the gut microbiota in humans and mice. In particular, ingestion of a high-fat (HF) diet reduces intestinal microbial diversity.

Marine algae are valuable sources of new bioactive materials and functional foods with potentially beneficial health effects [9]. Also, many studies reported that marine algae have the beneficial biological activities such as anti-inflammatory, antioxidant, anti-cancer, and anti-obesity properties [10,11,12,13]. *Codium fragile* (CF) is an edible green alga that is widely distributed in the coastal areas of Asia, Europe and Oceania. CF is often used as a cooking ingredient and has been used as an oriental medicine to treat intestinal and urinal disorders in Korea [14]. It has been reported that extracts of CF suppressed mouse ear edema and erythema in an animal model, and it also exhibited anti-allergic effects by inhibiting immunoglobulin E-sensitized granulation in RBL-2H3 cells [15]. It has been suggested that the anti-inflammatory effects of CF extracts are likely due to inhibition of NF-κB activity in mouse macrophages [16]. Moreover, beneficial effects of CF extracts include thrombolytic, anti-coagulant, anti-platelet, and immunostimulatory properties in mouse macrophages through activation of the NF-κB and the mitogen-activated protein kinase (MAPK) pathway closely related to cell proliferation and differentiation [17,18,19].

Recent study has reported that sulphated polysaccharide obtained from CF has a beneficial effect on improving diet-induced obesity [20]. While studies on the anti-obesity effect of CF extracts have been reported, mechanisms by which CF extracts improve obesity has not been investigated. Here, we evaluated the anti-obesity effects of CF extracts on gut microbiota of diet-induced obese mice model. Our findings suggest that the anti-obesity effects of CF extracts are largely due to the modulation of gut microbiota.

## 2. Materials and Methods

### 2.1. Preparation of Codium fragile (CF) Extracts

The CF was randomly collected from the coastal area of the island (Yaksan-do; 34°22′41.23″ N, 126°54′35.92″ E) in the southern coast of Korea (Figure S1) in August and September 2015 and then merged before washing process for removing salts. CF was repeatedly washed with fresh water for 10 min in order to remove salts and then air-dried at 50 °C for 72 h. Extraction was performed at 80 °C for 3 h using 60 times the amount of 70% ethanol (*v*/*v*). The CF extracts were then concentrated using a vacuum rotary evaporator. CF extracts were freeze-dried and stored at −20 °C. Composition of CF extract and extract condition optimization are summarized in Figure S2.

### 2.2. Animal Experiment

Specific pathogen-free 5-week-old male C57BL/6 mice were obtained from Daehan Biolink, Co., Ltd. (Eumsung, Chungbuk, Korea). The mice were acclimatized for at least 1 week prior to use, and were housed in an air-conditioned room with a 12 h light/dark cycle at a temperature of 22 ± 2 °C with 50 ± 5% relative humidity. The composition and formulation of the mouse diet AIN-93G and high-fat diet (Todobio, Gyeonggi, Korea) are detailed in Table S1. Water was provided to all animals ad-libitum. The mice were divided into three groups (*n* = 8 per group), the normal diet (Sham), high-fat diet (HF), and high-fat diet plus CF extracts (HFC). CF extracts were orally administrated daily (600 mg of CF extract per kg of body weight). Sham and HF group were given appropriate vehicles. Body weights were recorded once a week and blood glucose levels were measured by blood glucose meter (Accu-Chek Instant: Boehringer Mannheim, Seoul, Korea) at the end of the feeding trial. The animal experiments were conducted for 12 weeks. All experimental protocols for animal care were performed according to the rules and regulations of the Animal Ethics Committee of Jeju National University (the Institutional Animal Care and Use Committee of Jeju National University; Approval number 2016-0042) and conducted according to the Korean Animal and Plant Quarantine Agency guidelines (Laboratory Animal Guideline-75).

### 2.3. Histological Analysis

The epididymal white fat tissues were fixed in 10% buffered-neutral formalin. The fat sections were subjected to hematoxylin and eosin (H&E) staining to observe histopathological change (DKKorea, Seoul, Korea). An arbitrary scope was given to each microscopic field viewed at a magnification of 100×. Adipocyte size area was imaged using densitometry and analyzed using ImageJ software (National Institutes of Health, Bethesda, MD, USA).

### 2.4. Determination of Cholesterol Levels

Serum concentration of total cholesterol (T-C), low-density lipoprotein (LDL-C), and high-density lipoprotein (HDL-C) were analyzed at DKKorea (Seoul, Korea).

### 2.5. Gut Microbiota Analysis

Fresh fecal specimens were obtained from the mice at the end of the feeding trial. Fecal DNA was extracted using a QIAamp PowerFecal DNA Kit (Qiagen, Germantown, MD, USA) according to the manufacturer’s instructions. DNA concentrations were measured using a Qubit fluorometer (Invitrogen, Thermo Fisher Scientific, Waltham, MA, USA) and adjusted to 5 ng/μL using nuclease-free sterile water. The V4 hypervariable region of the 16S rRNA gene was amplified for microbial community analysis. MiSeq sequencing was performed at Macrogen (Seoul, Korea). MOTHUR software was used to remove the erroneous reads in accordance with MiSeq standard operating procedure (SOP) guidelines for the analysis process of 16S rRNA gene-based sequencing data (https://www.mothur.org/wiki/MiSeq_SOP) with a simple modification [21]. Briefly, paired-end reads were assembled using make.contigs and aligned to the SILVA database (version 132) contains ribosomal RNA (rRNA) gene sequences from the Bacteria, Archaea, and Eukaryota [22], singletons were removed using split.abund, chimeric sequences were detected using VSEARCH [23], and non-bacterial sequences were removed after taxonomic classification based on the Ribosomal Database Project (RDP) database (version 16) [24]. Operational taxonomic units (OTUs) were assigned using the opti.clust algorithm [25]. Number of reads per sample was set to 10,000 for downstream analyses. Species richness and evenness were estimated based on the Chao index [26] and Shannon index [27], respectively. Tree dendrograms and non-metric multidimensional scaling (NMDS) were calculated based on Bray-Curtis dissimilarity coefficients [28]. Metabolic pathways were predicted using Phylogenetic Investigation of Communities by Reconstruction of Unobserved States 2 (PICRUSt2) [29].

### 2.6. Statistical Analysis

The results are expressed as the means ± standard deviation (SD). Analysis of molecular variance (AMOVA) was used to evaluate significant differences based on NMDS coordinates. Ellipses in NMDS were drawn with a 0.95 confidence level using R vegan package. AMOVA was used to estimate significant difference of the gut microbiota in NMDS. Statistical significance was accepted for *p* values <0.001. The taxonomic composition of gut microbiota and OTUs with significantly differential abundance were identified using linear discriminant analysis (LDA) effect size (LEfSe) software [30]. ALDEx2 is a differential abundance analysis package in R software and was used to identify significantly different predicted metabolic pathways [31]. Spearman rank correlation analysis was performed to investigate associations between OTUs and predicted metabolic pathways detected in ALDEx2 analysis. Statistically significant differences within the parameters and ecological indices were tested using analysis of variance (ANOVA) with a Tukey-Kramer test. Statistical significance was accepted for *p* values <0.05.

## 3. Results

### 3.1. CF Extracts Reduce the High-Fat Diet (HF)-Induced Fat Deposition in Mice

**Figure 1:**
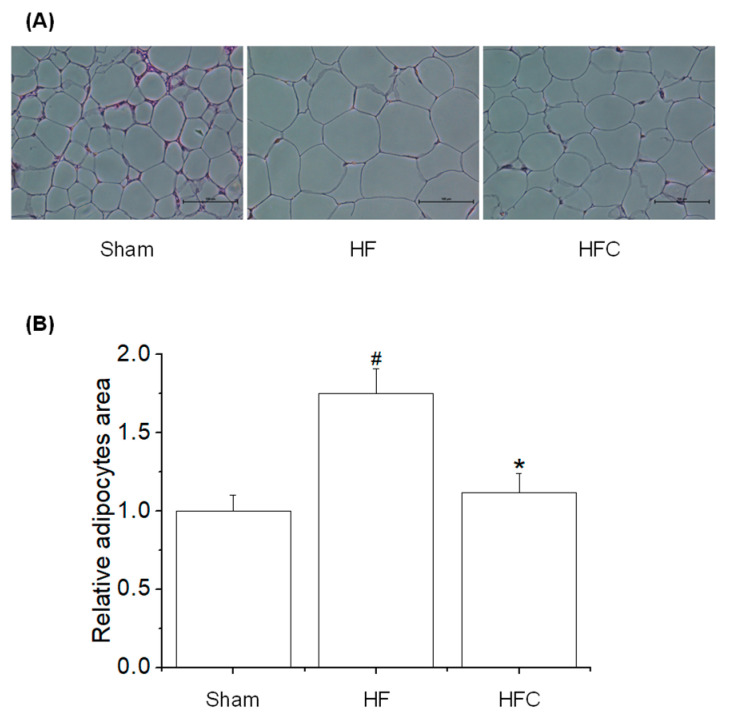
Effect of CF extracts on the feature indexes of obesity in HF-fed mice. (**A**) Hematoxylin and eosin (H&E) staining of the epididymal white adipocytes tissue and (**B**) The adipocyte size. All results are expressed as the means ± SD (*n* = 8). Compared with Sham group, # *p* < 0.05, Compared with HF group, * *p* < 0.05. Sham, HF and HFC indicated normal diet-fed group, high-fat diet group, and high-fat diet plus *Codium fragile* (CF) extracts-fed group, respectively.

We investigated the effects of CF extracts on gut microbiota in HF-fed mice. The mice were treated with CF extracts for 12 weeks. We examined the inhibitory effects of CF extracts on body weight gain and fat deposition in mice. As shown in Table 1, CF extracts significantly decreased the weight of mice compared to those of HF-fed mice. We evaluated the composition of fat after sacrificing mice at the end of the feeding trial. The pathological changes in adipocyte tissues in all mice are shown in Figure 1 HF-fed mice with or without CF extracts showed increased adipocyte size compared to Sham. On the other hand, the CF extracts showed decreased adipocyte size compared to that of HF-fed mice (Figure 1). These results suggest that CF extracts could suppress the HF-induced body weight gain by improving the adipocyte hyperplasia and hypertrophy.

### 3.2. CF Extracts Suppress the HF-Induced Lipid Level in Mice

As fat accumulation is the main pathological feature of obesity, serum lipid level was evaluated in mice. The levels of T-C and LDL-C were increased in mice fed with HF. The levels of T-C and LDL-C were significantly decreased by treatment with CF extracts at a daily dose of 600 mg/kg. However, the level of HDL-C are not significantly different between the HF and the HFC groups (Table 2). Furthermore, the glucose concentration was significantly suppressed in the HFC group compared with the HF group. These results suggest that CF extracts could suppress the HF-induced body weight gain via improving of the fat accumulation.

### 3.3. Comparison Analysis of Mice Gut Microbiota According to the Types of Treatment

**Figure 2:**
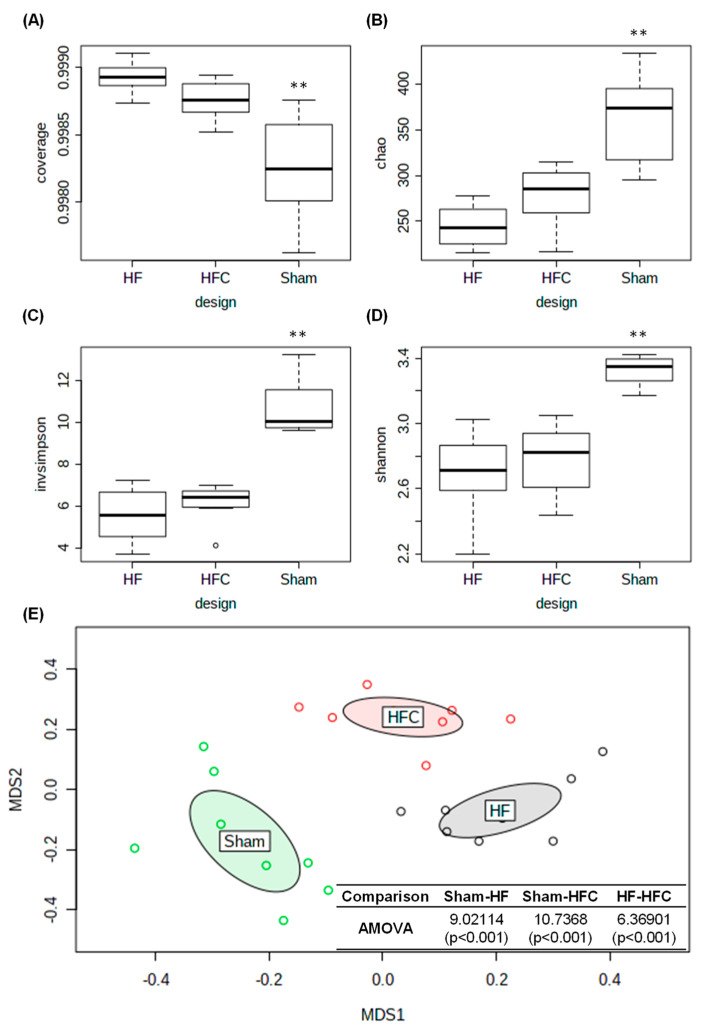
The comparison analysis of gut microbiota using ecological indices and non-metric multidimensional scaling (NMDS). (**A**) Comparison of coverage, (**B**) species richness, (**C**) Simpson diversity, (**D**) Shannon diversity, and (**E**) NMDS plotting combined with analysis of molecular variance (AMOVA). All results are expressed as the means ± SD (*n* = 8). Sham, HF and HFC indicated normal diet-fed group, high-fat diet group and high-fat diet plus *Codium fragile* (CF) extracts-fed group, respectively. ** indicates significant difference compared to HF and HFC (*p* < 0.01).

To investigate the effect of CF extracts on the regulation of the intestinal microbial total structure, we performed high-throughput sequencing using the Illumina MiSeq platform. We obtained 1,285,868 high-quality and valid sequences in this study. The number of reads for each sample was adjusted to 10,000 prior to the downstream analyses, while maintaining greater than 99% coverage (Figure 2A). In this study, species richness and evenness were not significantly different between the HF and HFC groups of mice while all ecological indices in Sham group were significantly higher compared to those of high-fat diet groups (Figure 2B–D). Figure 2E also showed that the Sham gut microbiota was significantly different from high-fat diet groups. These results indicate that high-fat diet induced intestinal dysbiosis in the mice gut microbiota. To investigate the anti-obesity effects of CF extract, we compared gut microbiota between HF and HFC groups.

### 3.4. CF Extracts Ameliorate the HF-Induced Dysbiosis of the Gut Microbiota in Mice

**Figure 3:**
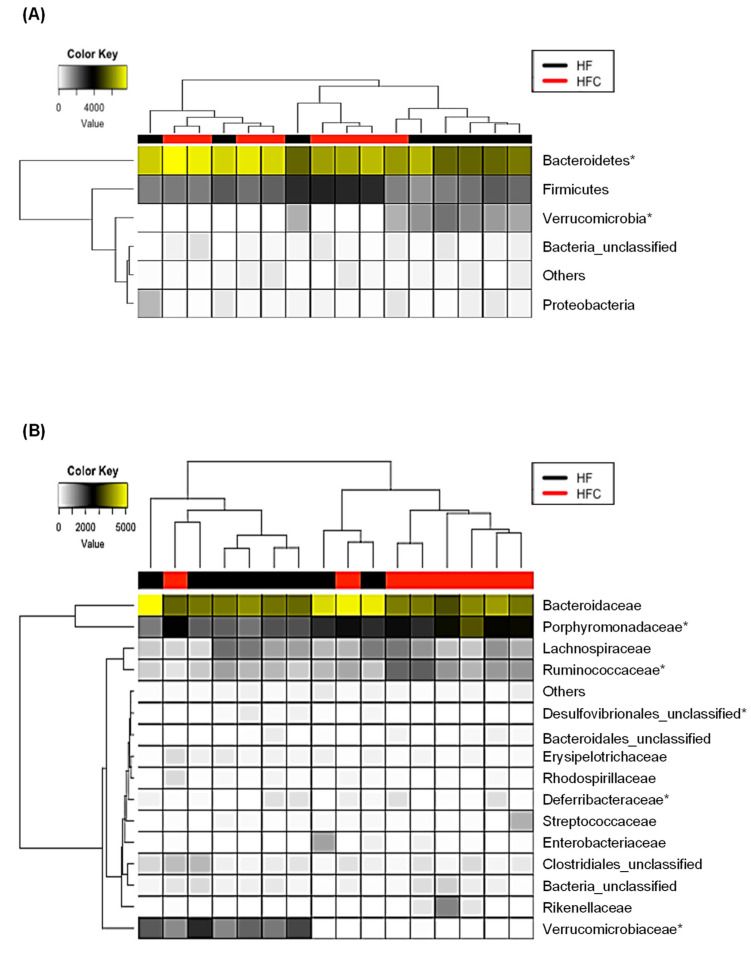

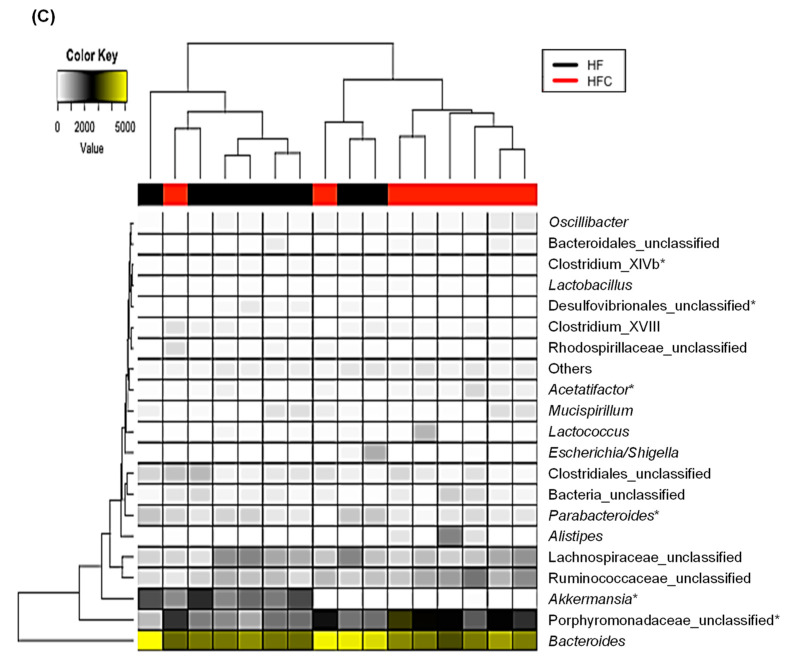
Effect of CF extracts on the taxonomic composition of gut microbiota in HF-fed mice. (**A**) Phylum, (**B**) family, and (**C**) genus levels. All results are expressed as the means ± SD (*n* = 8). * Indicates significantly difference (*p* < 0.05). HF and HFC indicated high-fat diet group and high-fat diet plus *Codium fragile* (CF) extracts-fed group, respectively.

**Figure 4:**
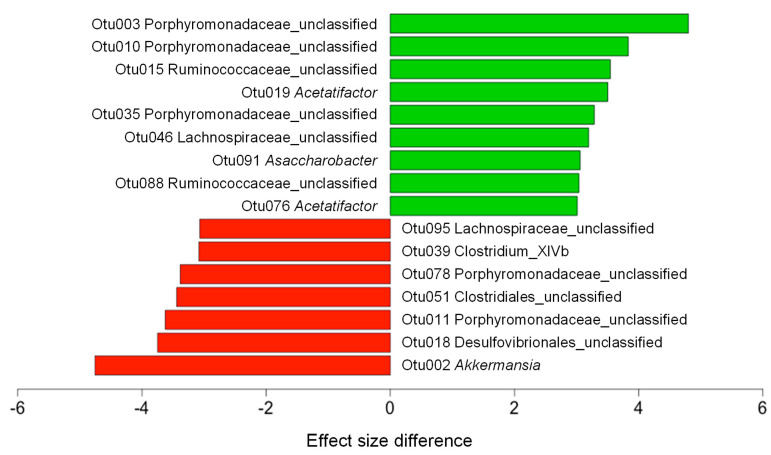
Effect of CF extracts on the comparison of gut microbiota in HF-fed mice. Abundance analysis was performed using the linear discriminant analysis effect size (LEfSe) (red: HF, green: HFC). HF and HFC indicated high-fat diet group and high-fat diet plus *Codium fragile* (CF) extracts-fed group, respectively.

**Figure 5:**
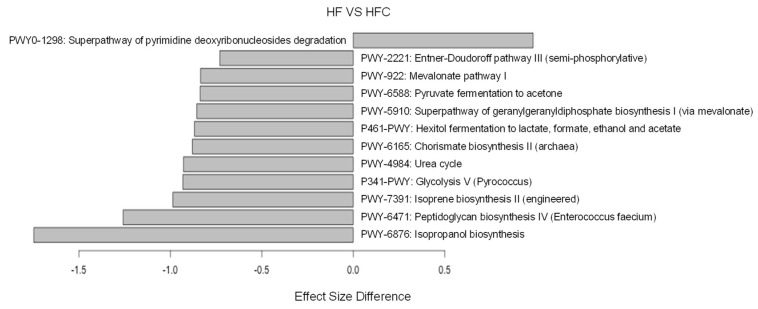
Differentially enriched predicted metabolic pathways identified by ALDEx2 (*p* < 0.05). Negative and positive effect size indicate significantly depleted and enriched predicted metabolic pathways in the HFC group in comparison to the HF group, respectively. HF and HFC indicate high-fat diet group and high-fat diet plus *Codium fragile* (CF) extracts group, respectively.

To check if CF extracts changed the taxonomic composition of gut microbiota, the relative abundance at the phylum, family, and genus levels were examined using LEfSe analysis. At the phylum level, the CF extracts increased the relative abundance of Bacteroidetes and reduced the relative abundance of Verrucomicrobia (Figure 3A). The increase in Bacteroidetes was largely due to the increase in the families of Porphyromonadaceae and Ruminococcaceae, while the decrease in Verrucomicrobia was due to a loss of the genus *Akkermansia* (Figure 3B,C). To further explain the changes in the gut microbiota at the lower taxonomic levels, we examined the gut microbiota of the HF and HFC using LEfSe analysis. CF extracts significantly shifted the gut microbiota composition by increasing members of the family Porphyromonadaceae and genus *Acetatifactor*, while decreasing the abundance of other members of Porphyromonadaceae and the genus *Akkermansia* (Figure 4). Also, we performed ALDEx2 analysis to identify differentially enriched predicted intestinal metabolic pathways. As shown in Figure 5, CF extracts enriched one predicted metabolic pathway (PWY0-1298: Superpathway of pyrimidine deoxyribonucleosides degradation), which has been reported to modulate bacterial colonization capabilities in the gut [32]. On the other hand, there were 11 pathways that were significantly decreased by supplementing CF extracts. Among them, some have been previously reported as obesity biomarkers. For example, mevalonate is involved in cholesterol synthesis and is essential for adipocyte survival, and isoprene biosynthesis has also been reported as a potential obesity marker [33,34]. Moreover, decreases in pyruvate fermentation and glycolysis may indicate the decrease in energy harvesting. Reduction of the tryptophan precursor chorismate leads to lower production of serotonin in the gut. While serotonin is a hormone that controls appetite, a recent study showed that nearly 90% of serotonin is peripheral serotonin that works as an energy-saving hormone, suggesting that a reduction in chorismate biosynthesis may be related to its anti-obesity effect [35]. Urea metabolism has also been reported to be associated with obesity in adults and peptidoglycan biosynthesis is involved in producing bacterial lipopolysaccharides, which may cause obesity if it enters into intestinal capillaries [36,37]. These results suggest that CF extracts reduced these obesity-related predicted intestinal metabolic pathways.

Furthermore, we investigated the association of bacterial taxa with obesity-related predicted intestinal metabolic pathways with Spearman’s rank correlation analysis. Table 3 shows the significant correlation to between the LEfSe-selected OTUs and obesity-related predicted intestinal metabolic pathways. Our results show that the increase in abundance of one unclassified genus of the family Porphyromonadaceae was positively associated with pyrimidine degradation. Meanwhile, the increase in *Acetatifactor* was negatively associated with predicted metabolic pathways for the biosynthesis of isoprene, chorismite, and geranylgeranyl diphosphate. Unknown genera of the families Porphyromonadaceae, Lachnospiraceae, and Ruminococcaceae also showed associations with the remaining predicted metabolic pathways. It should be noted that CF extracts significantly decreased the abundance of *Akkermansia*, which was positively associated with isoprene biosynthesis.

## 4. Discussion

Obesity is an increase in body weight due to excessive accumulation of fat tissue, which contributes to an increased prevalence of obesity-related metabolic dysfunction [38]. It has been known that eating foods rich in dietary fiber components, such as fruits and vegetables, can have beneficial effects on anti-obesity and obesity-related metabolic diseases [39,40,41]. Previous study reported that sulphated polysaccharide obtained from *Codium fragile* (CFSP) decreased the HF-induced body weight gain via inhibition of lipid accumulation in a rat model. Furthermore, CFSP restored the antioxidant enzymes (superoxide dismutase, catalase, and glutathione peroxidase) reduced by HF and inhibited the thiobarbituric acid reactive substances [20]. We also observed that daily supplementation with *Codium fragle* (CF) extracts could significantly decreased body weight via suppression of fat accumulation in HF-fed mice. We also investigated anti-obesity effects of CF extracts on HF-fed mice through gut microbiota modulation along with the changes of predicted intestinal metabolic pathways.

Recent studies have shown that changes of gut microbiota act as important influencing factors that contribute to the development of obesity, insulin resistance, and inflammation [42,43,44]. CF extracts caused the significant reduction of body weights, size of adipocytes, serum cholesterol, and blood glucose levels in HF-induced obese mice. It has been reported that transplanting feces of metformin-treated patients lowered the serum glucose level in mice, suggesting that gut microbiota plays a key role in controlling the serum glucose levels [45,46]. In addition, gut microbiota also stimulates secretion of glucagon-like peptide-1 (GLP-1), which has anti-inflammatory effects on adipocytes and macrophage infiltration by increasing insulin sensitivity in diabetic mice [47]. Recently, Martin et al. [48] reported that gut microbiota regulates glucose homeostasis through serotonin synthesis in enterochromaffin cells. In this study, we observed a significant reduction of chorismate biosynthesis II, which is a precursor of tryptophan, which thereby consequently reduces the amount of serotonin. We also observed increased abundance of the genus *Acetatifactor*, which was associated with the reduced chorismite biosynthesis. The genus *Acetatifactor* has been also reported to activate bile acid membrane receptor TGR5 and stimulate GLP-1 secretion, which ultimately improves liver function and tolerance to insulin and glucose [49]. These results indicate that modulating gut microbiota with CF extracts might be considered to be a therapeutic strategy against HF-induced metabolic syndromes.

Several plants with bioactive ingredients, such as bamboo-shaving, *Angelica keiskei*, and citrus peel, reduced body weight gain and ameliorated lipid metabolic disorders by altering the gut microbiota [50]. In this study, we have investigated how anti-obesity effects of CF extracts is associated with modulation of gut microbiota. We observed that CF extracts increased the ratio of Bacteroidetes/Firmicutes in HF-fed mice, indicating that CF extracts shifted the gut microbiota toward the lean type [51]. *Akkermansia* has been known its anti-obesity effects in humans and animals [52,53,54,55]. However, it has also been reported that *Akkermansia* causes intestinal inflammation by devouring the mucin layers when mice are fed fiber-free diet for a long term [56]. In addition, inflammatory bowel disease (IBD) and type 2 diabetes have been reported to be associated with higher abundance of *Akkermansia*, which is reversed by eating seaweed extract [57,58,59]. These results suggest that *Akkermansia* exhibits diverse responses according to host conditions, which emphasizes the need for further clarification of the roles of *Akkermansia* in obesity.

Among the metabolic shifts caused by CF extract, we observed the genus *Acetatifactor* was most frequently involved. Currently, there is only a single species of *Acetatifactor* reported, which is known to produce acetate and butyrate in the gut [60]. Changes in the gut microbiota affect production of luminal short-chain fatty acids (SCFAs), major metabolites including acetic acid, propionic acid, and butyric acid [61]. Many studies shown that the SCFAs regulate weight management and insulin sensitivity as well as prevent HF-induced metabolic shifts [62,63,64,65]. *Acetatifactor* is also reported to decrease with consumption of high-fat and high-calorie diets, while it increases with the oral administration of *Lactobacillus* as well as injection of extracts from the plant *Cinnamonmi ramulus* [66,67]. In addition, the abundance of *Acetatifactor* has been reported to be associated with bile acids [68] which is synthesized from LDL-C in the liver and solubilizes dietary fats to allow their intestinal absorption. Taken together, the increase of *Acetatifactor* may play a key role in the anti-obesity effect.

Furthermore, CF extracts significantly increased the relative abundance of some OTUs classified in the families Ruminococcaceae and Lachnospiraceae in diet-induced obese mice. Previous study reported that fucoidan from *Laminaria japonica* significantly increased the relative abundance of Ruminococcaceae while the abundance of Verrucomicrobia decreased, which is consistent with our current results [69]. Ruminococcaceae and Lachnospiraceae are predominant families of intestinal bacteria involved in carbohydrate metabolisms [70,71]. Many SCFA-producing bacteria also belong to these families. In addition, we observed that CF extracts increased the members of the family Porphyromonadaceae which includes SCFA-producing bacteria such as *Butyricimonas*, *Coprobacter*, and *Macellibacteroides* [72,73,74]. These results suggest that CF extracts increase the abundance of SCFA producers, contributing to its anti-obesity effect.

## 5. Conclusions

In conclusion, the present study suggest that CF extracts may have an anti-obesity effect, the mechanisms of which likely involve modulation of gut microbiota. The modulated gut microbiota are positively associated with a depleted predicted metabolic pathway related to obesity. Therefore, we conclude that CF extracts have the potential to be used as an anti-obesity functional food.

## Figures and Tables

**Table 1:** The change of body weight in three groups.

	Sham	HF	HFC
Initial Body Weight (g)	20.3 ± 0.81	20.0 ± 1.22	19.5 ± 1.53
Final Body Weight (g)	27.4 ± 1.47	36.2 ± 3.89 #	30.8 ± 2.75 *
Body Weight Change (g)	7.1 ± 0.77	16.2 ± 2.79 #	11.3 ± 2.47 *

Data were expressed as mean ± standard deviation (SD, *n* = 8). Different lowercase letters indicated significant differences between three groups. Compared with Sham group, # *p* < 0.01, Compared with HF group, * *p* < 0.05. Sham, HF and HFC indicated normal diet-fed group, high-fat diet group, and high-fat diet plus *Codium fragile* (CF) extracts-fed group, respectively.

**Table 2:** The level of serum lipid and glucose in three groups.

	Sham	HF	HFC
T-C (mg/dL)	156.8 ± 15.43	208.3 ± 14.34	186.0 ± 21.62
HDL-C (mg/dL)	98.5 ± 6.26	100.6 ± 8.55	97.0 ± 8.92
LDL-C (mg/dL)	17.5 ± 2.50	28.8 ± 2.59	18.8 ± 3.96 *
Glucose (mg/dL)	96.4 ± 11.48	128.4 ± 3.38	103.0 ± 7.77 *

Data were expressed as mean ± SD (*n* = 8). Different lowercase letters indicated significant differences between three groups. Compared with HF group, * *p* < 0.05. Sham, HF and HFC indicated normal diet-fed group, high-fat diet group, and high-fat diet plus *Codium fragile* (CF) extracts-fed group, respectively.

**Table 3:** Biomarker operational taxonomic units (OTUs) associated with the metabolic shifts caused by *Codium fragile* (CF) extracts. The associated biomarker OTUs were selected by *q* < 0.05 and *p* < 0.05 calculated using Spearman’s rank correlation analysis.

Pathway	Increased/Decreased	Description	Associated Biomarker OTUs (Spearman Coef.)
PWY0-1298	Increased	Pyrimidine deoxyribonucleosides degradation	Otu011: Porphyromonadaceae_unclassified (0.79)
PWY-6165	Decreased	Chorismate biosynthesis II	Otu086: *Acetatifactor* (−0.76) Otu124 *: Ruminococceae_unclassified (0.73)
PWY-7391	Decreased	Isoprene biosynthesis II	Otu157: Porphyromonadaceae_unclassified (−0.99) Otu120: Lachnospiraceae_unclassified (−0.81) Otu146: *Acetatifactor* (−0.81) Otu076: *Acetatifactor* (−0.71) Otu002 *: *Akkermansia* (0.77)
PWY-922	Decreased	Mevalonate pathway I	Otu123: Ruminococcaceae_unclassified (−0.76) Otu124 *: Ruminococcaceae_unclassified (0.82)
PWY-5910	Decreased	Geranylgeranyldiphosphate biosynthesis I	Otu136: *Acetatifactor* (−0.77) Otu175: Lachnospiraceae_unclassified (−0.73) Otu173: *Acetatifactor* (−0.71) Otu068 *: Lachnospiraceae_unclassified (0.73) Otu124 *: Ruminococcaceae_unclassified (0.82)
P461-PWY	Decreased	Hexitol fermentation to lactate, formate, ethanol and acetate	Otu039 *: Clostridium_XlVb (0.73)
PWY-2221	Decreased	Enter-Doudoroff pathway III	Otu123: Ruminococcaceae_unclassified (−0.81) Otu124 *: Ruminococcaceae_unclassified (0.81)
PWY-6588	Decreased	Pyruvate fermentation to acetone	Otu010: Porphyromonadaceae_unclassified (−0.77) Otu111: Lachnospiraceae_unclassified (−0.74)

* Indicates significantly decreased abundance by CF extracts.

## Supplementary Materials

The following are available online at https://www.mdpi.com/2072-6643/12/6/1848/s1: Figure S1: The *Codium fragile* (CF) sampling location (Yaksan-do; 34°22’41.23"N, 126°54’35.92"E), Figure S2: The extraction yield of CF extracts depending on both extraction time and extraction temperature (**A**) and the composition analysis of CF extracts (**B**), Figure S3: The clustering analysis of the gut microbiota using tree dendrogram based on braycurtis dissimilarity, and Table S1: The compositions and formulas of chow diet and high-fat diet.

## References

[1] De Ferranti S., Mozaffarian D. (2008). The perfect storm: Obesity, adipocyte dysfunction, and metabolic consequences. Clin. Chem..

[2] Peyton K.J., Liu X.M., Shebib A.R., Johnson F.K., Johnson R.A., Durante W. (2018). Arginase inhibition prevents the development of hypertension and improves insulin resistance in obese rats. Amino Acids.

[3] Cox A.J., West N.P., Cripps A.W. (2015). Obesity, inflammation, and the gut microbiota. Lancet Diabetes Endocrinol..

[4] Zhao L., Wang Y., Zhang G., Zhang T., Lou J., Liu J. (2019). L-Arabinose Elicits Gut-Derived Hydrogen Production and Ameliorates Metabolic Syndrome in C57BL/6J Mice on High-Fat-Diet. Nutrients.

[5] Le Chatelier E., Nielsen T., Qin J., Prifti E., Hildebrand F., Falony G., Almeida M., Arumugam M., Batto J.M., Kennedy S. (2013). Richness of human gut microbiome correlates with metabolic markers. Nature.

[6] Jang H.M., Han S.K., Kim J.K., Oh S.J., Jang H.B., Kim D.H. (2019). Lactobacillus sakei Alleviates High-Fat-Diet-Induced Obesity and Anxiety in Mice by Inducing AMPK Activation and SIRT1 Expression and Inhibiting Gut Microbiota-Mediated NF-kappaB Activation. Mol. Nutr. Food Res..

[7] Sekirov I., Russell S.L., Antunes L.C., Finlay B.B. (2010). Gut microbiota in health and disease. Physiol. Rev..

[8] Chen Y., Jin L., Li Y., Xia G., Chen C., Zhang Y. (2018). Bamboo-shaving polysaccharide protects against high-diet induced obesity and modulates the gut microbiota of mice. J. Funct. Foods.

[9] Perez M.J., Falque E., Dominguez H. (2016). Antimicrobial Action of Compounds from Marine Seaweed. Mar. Drugs.

[10] Su J., Guo K., Huang M., Liu Y., Zhang J., Sun L., Li D., Pang K.L., Wang G., Chen L. (2019). Fucoxanthin, a Marine Xanthophyll Isolated From Conticribra weissflogii ND-8: Preventive Anti-Inflammatory Effect in a Mouse Model of Sepsis. Front. Pharmacol..

[11] Yayeh T., Im E.J., Kwon T.H., Roh S.S., Kim S., Kim J.H., Hong S.B., Cho J.Y., Park N.H., Rhee M.H. (2014). Hemeoxygenase 1 partly mediates the anti-inflammatory effect of dieckol in lipopolysaccharide stimulated murine macrophages. Int. Immunopharmacol..

[12] Khalifa S.A.M., Elias N., Farag M.A., Chen L., Saeed A., Hegazy M.F., Moustafa M.S., Abd El-Wahed A., Al-Mousawi S.M., Musharraf S.G. (2019). Marine Natural Products: A Source of Novel Anticancer Drugs. Mar. Drugs.

[13] Wan-Loy C., Siew-Moi P. (2016). Marine Algae as a Potential Source for Anti-Obesity Agents. Mar. Drugs.

[14] Sanjeewa K.K.A., Lee W., Jeon Y.-J. (2018). Nutrients and bioactive potentials of edible green and red seaweed in Korea. Fish. Aquat. Sci..

[15] Kimiya T., Ohtani K., Satoh S., Abe Y., Ogita Y., Kawakita H., Hamada H., Konishi Y., Kubota S., Tominaga A. (2008). Inhibitory effects of edible marine algae extracts on degranulation of RBL-2H3 cells and mouse eosinophils. Fish. Sci..

[16] Lee S.A., Moon S.M., Choi Y.H., Han S.H., Park B.R., Choi M.S., Kim J.S., Kim Y.H., Kim D.K., Kim C.S. (2017). Aqueous extract of Codium fragile suppressed inflammatory responses in lipopolysaccharide-stimulated RAW264.7 cells and carrageenan-induced rats. Biomed. Pharmacother..

[17] Kim J.E., Monmai C., Rod-In W., Jang A.Y., You S.G., Lee S.M., Park W.J. (2019). Immune Enhancement Effects of Codium fragile Anionic Macromolecules Combined with Red Ginseng Extract in Immune-Suppressed Mice. J. Microbiol. Biotechnol..

[18] Choi J.H., Sapkota K., Park S.E., Kim S., Kim S.J. (2013). Thrombolytic, anticoagulant and antiplatelet activities of codiase, a bi-functional fibrinolytic enzyme from Codium fragile. Biochimie.

[19] Tabarsa M., Karnjanapratum S., Cho M., Kim J.K., You S. (2013). Molecular characteristics and biological activities of anionic macromolecules from Codium fragile. Int. J. Biol. Macromol..

[20] Kolsi R.B.A., Jardak N., Hajkacem F., Chaaben R., Jribi I., Feki A.E., Rebai T., Jamoussi K., Fki L., Belghith H. (2017). Anti-obesity effect and protection of liver-kidney functions by Codium fragile sulphated polysaccharide on high fat diet induced obese rats. Int. J. Biol. Macromol..

[21] Schloss P.D., Westcott S.L., Ryabin T., Hall J.R., Hartmann M., Hollister E.B., Lesniewski R.A., Oakley B.B., Parks D.H., Robinson C.J. (2009). Introducing mothur: Open-source, platform-independent, community-supported software for describing and comparing microbial communities. Appl. Environ. Microbiol..

[22] Quast C., Pruesse E., Yilmaz P., Gerken J., Schweer T., Yarza P., Peplies J., Glockner F.O. (2013). The SILVA ribosomal RNA gene database project: Improved data processing and web-based tools. Nucleic Acids Res..

[23] Rognes T., Flouri T., Nichols B., Quince C., Mahe F. (2016). VSEARCH: A versatile open source tool for metagenomics. PeerJ.

[24] Cole J.R., Chai B., Farris R.J., Wang Q., Kulam-Syed-Mohideen A.S., McGarrell D.M., Bandela A.M., Cardenas E., Garrity G.M., Tiedje J.M. (2007). The ribosomal database project (RDP-II): Introducing myRDP space and quality controlled public data. Nucleic Acids Res..

[25] Westcott S.L., Schloss P.D. (2017). OptiClust, an Improved Method for Assigning Amplicon-Based Sequence Data to Operational Taxonomic Units. MSphere.

[26] Chao A., Chazdon R.L., Colwell R.K., Shen T.-J. (2004). A new statistical approach for assessing similarity of species composition with incidence and abundance data. Ecol. Lett..

[27] Shannon C.E. (1997). The mathematical theory of communication 1963. MD Comput..

[28] Beals E.W. (1984). Bray-Curtis Ordination: An Effective Strategy for Analysis of Multivariate Ecological Data. Adv. Ecol. Res..

[29] Douglas G.M., Maffei V.J., Zaneveld J., Yurgel S.N., Brown J.R., Taylor C.M., Huttenhower C., Langille M.G.I. (2020). PICRUSt2: An improved and customizable approach for metagenome inference. bioRxiv.

[30] Segata N., Izard J., Waldron L., Gevers D., Miropolsky L., Garrett W.S., Huttenhower C. (2011). Metagenomic biomarker discovery and explanation. Genome Biol..

[31] Fernandes A.D., Reid J.N., Macklaim J.M., McMurrough T.A., Edgell D.R., Gloor G.B. (2014). Unifying the analysis of high-throughput sequencing datasets: Characterizing RNA-seq, 16S rRNA gene sequencing and selective growth experiments by compositional data analysis. Microbiome.

[32] Vogel-Scheel J., Alpert C., Engst W., Loh G., Blaut M. (2010). Requirement of purine and pyrimidine synthesis for colonization of the mouse intestine by *Escherichia coli*. Appl. Environ. Microbiol..

[33] Yeh Y.S., Jheng H.F., Iwase M., Kim M., Mohri S., Kwon J., Kawarasaki S., Li Y., Takahashi H., Ara T. (2018). The Mevalonate Pathway Is Indispensable for Adipocyte Survival. iScience.

[34] Alkhouri N., Eng K., Cikach F., Patel N., Yan C., Brindle A., Rome E., Hanouneh I., Grove D., Lopez R. (2015). Breathprints of childhood obesity: Changes in volatile organic compounds in obese children compared with lean controls. Pediatr. Obes..

[35] Namkung J., Kim H., Park S. (2015). Peripheral Serotonin: A New Player in Systemic Energy Homeostasis. Mol. Cells.

[36] Cao Y.F., Li J., Zhang Z., Liu J., Sun X.Y., Feng X.F., Luo H.H., Yang W., Li S.N., Yang X. (2019). Plasma Levels of Amino Acids Related to Urea Cycle and Risk of Type 2 Diabetes Mellitus in Chinese Adults. Front. Endocrinol..

[37] Taira R., Yamaguchi S., Shimizu K., Nakamura K., Ayabe T., Taira T. (2015). Bacterial cell wall components regulate adipokine secretion from visceral adipocytes. J. Clin. Biochem. Nutr..

[38] Barathikannan K., Chelliah R., Rubab M., Daliri E.B., Elahi F., Kim D.H., Agastian P., Oh S.Y., Oh D.H. (2019). Gut Microbiome Modulation Based on Probiotic Application for Anti-Obesity: A Review on Efficacy and Validation. Microorganisms.

[39] Lattimer J.M., Haub M.D. (2010). Effects of dietary fiber and its components on metabolic health. Nutrients.

[40] Dreher M.L. (2018). Whole Fruits and Fruit Fiber Emerging Health Effects. Nutrients.

[41] Hervik A.K., Svihus B. (2019). The Role of Fiber in Energy Balance. J. Nutr. Metab..

[42] Liu Z., Wang N., Ma Y., Wen D. (2019). Hydroxytyrosol Improves Obesity and Insulin Resistance by Modulating Gut Microbiota in High-Fat Diet-Induced Obese Mice. Front. Microbiol..

[43] Sikalidis A.K., Maykish A. (2020). The Gut Microbiome and Type 2 Diabetes Mellitus: Discussing a Complex Relationship. Biomedicines.

[44] Lee S., Keirsey K.I., Kirkland R., Grunewald Z.I., Fischer J.G., de La Serre C.B. (2018). Blueberry Supplementation Influences the Gut Microbiota, Inflammation, and Insulin Resistance in High-Fat-Diet-Fed Rats. J. Nutr..

[45] Gerard C., Vidal H. (2019). Impact of Gut Microbiota on Host Glycemic Control. Front. Endocrinol..

[46] Wu H., Esteve E., Tremaroli V., Khan M.T., Caesar R., Manneras-Holm L., Stahlman M., Olsson L.M., Serino M., Planas-Felix M. (2017). Metformin alters the gut microbiome of individuals with treatment-naive type 2 diabetes, contributing to the therapeutic effects of the drug. Nat. Med..

[47] Lee Y.S., Park M.S., Choung J.S., Kim S.S., Oh H.H., Choi C.S., Ha S.Y., Kang Y., Kim Y., Jun H.S. (2012). Glucagon-like peptide-1 inhibits adipose tissue macrophage infiltration and inflammation in an obese mouse model of diabetes. Diabetologia.

[48] Martin A.M., Yabut J.M., Choo J.M., Page A.J., Sun E.W., Jessup C.F., Wesselingh S.L., Khan W.I., Rogers G.B., Steinberg G.R. (2019). The gut microbiome regulates host glucose homeostasis via peripheral serotonin. Proc. Natl. Acad. Sci. USA.

[49] Pathak P., Xie C., Nichols R.G., Ferrell J.M., Boehme S., Krausz K.W., Patterson A.D., Gonzalez F.J., Chiang J.Y.L. (2018). Intestine farnesoid X receptor agonist and the gut microbiota activate G-protein bile acid receptor-1 signaling to improve metabolism. Hepatology.

[50] Tung Y.C., Chang W.T., Li S., Wu J.C., Badmeav V., Ho C.T., Pan M.H. (2018). Citrus peel extracts attenuated obesity and modulated gut microbiota in mice with high-fat diet-induced obesity. Food Funct..

[51] Ley R.E., Backhed F., Turnbaugh P., Lozupone C.A., Knight R.D., Gordon J.I. (2005). Obesity alters gut microbial ecology. Proc. Natl. Acad. Sci. USA.

[52] Plovier H., Everard A., Druart C., Depommier C., Van Hul M., Geurts L., Chilloux J., Ottman N., Duparc T., Lichtenstein L. (2017). A purified membrane protein from Akkermansia muciniphila or the pasteurized bacterium improves metabolism in obese and diabetic mice. Nat. Med..

[53] Depommier C., Everard A., Druart C., Plovier H., Van Hul M., Vieira-Silva S., Falony G., Raes J., Maiter D., Delzenne N.M. (2019). Supplementation with Akkermansia muciniphila in overweight and obese human volunteers: A proof-of-concept exploratory study. Nat. Med..

[54] Geerlings S.Y., Kostopoulos I., de Vos W.M., Belzer C. (2018). Akkermansia muciniphila in the Human Gastrointestinal Tract: When, Where, and How?. Microorganisms.

[55] Parada Venegas D., De la Fuente M.K., Landskron G., Gonzalez M.J., Quera R., Dijkstra G., Harmsen H.J.M., Faber K.N., Hermoso M.A. (2019). Short Chain Fatty Acids (SCFAs)-Mediated Gut Epithelial and Immune Regulation and Its Relevance for Inflammatory Bowel Diseases. Front. Immunol..

[56] Desai M.S., Seekatz A.M., Koropatkin N.M., Kamada N., Hickey C.A., Wolter M., Pudlo N.A., Kitamoto S., Terrapon N., Muller A. (2016). A Dietary Fiber-Deprived Gut Microbiota Degrades the Colonic Mucus Barrier and Enhances Pathogen Susceptibility. Cell.

[57] Seregin S.S., Golovchenko N., Schaf B., Chen J., Pudlo N.A., Mitchell J., Baxter N.T., Zhao L., Schloss P.D., Martens E.C. (2017). NLRP6 Protects Il10(-/-) Mice from Colitis by Limiting Colonization of Akkermansia muciniphila. Cell Rep..

[58] Qin J., Li Y., Cai Z., Li S., Zhu J., Zhang F., Liang S., Zhang W., Guan Y., Shen D. (2012). A metagenome-wide association study of gut microbiota in type 2 diabetes. Nature.

[59] Yan X., Yang C., Lin G., Chen Y., Miao S., Liu B., Zhao C. (2019). Antidiabetic Potential of Green Seaweed Enteromorpha prolifera Flavonoids Regulating Insulin Signaling Pathway and Gut Microbiota in Type 2 Diabetic Mice. J. Food Sci..

[60] Pfeiffer N., Desmarchelier C., Blaut M., Daniel H., Haller D., Clavel T. (2012). Acetatifactor muris gen. nov., sp. nov., a novel bacterium isolated from the intestine of an obese mouse. Arch. Microbiol..

[61] Feng W., Ao H., Peng C. (2018). Gut Microbiota, Short-Chain Fatty Acids, and Herbal Medicines. Front. Pharmacol..

[62] Den Besten G., Bleeker A., Gerding A., van Eunen K., Havinga R., van Dijk T.H., Oosterveer M.H., Jonker J.W., Groen A.K., Reijngoud D.J. (2015). Short-Chain Fatty Acids Protect Against High-Fat Diet-Induced Obesity via a PPARgamma-Dependent Switch From Lipogenesis to Fat Oxidation. Diabetes.

[63] Lu Y., Fan C., Li P., Lu Y., Chang X., Qi K. (2016). Short Chain Fatty Acids Prevent High-fat-diet-induced Obesity in Mice by Regulating G Protein-coupled Receptors and Gut Microbiota. Sci. Rep..

[64] Chambers E.S., Morrison D.J., Frost G. (2015). Control of appetite and energy intake by SCFA: What are the potential underlying mechanisms?. Proc. Nutr. Soc..

[65] Weitkunat K., Stuhlmann C., Postel A., Rumberger S., Fankhanel M., Woting A., Petzke K.J., Gohlke S., Schulz T.J., Blaut M. (2017). Short-chain fatty acids and inulin, but not guar gum, prevent diet-induced obesity and insulin resistance through differential mechanisms in mice. Sci. Rep..

[66] Dong T.S., Chang H.H., Hauer M., Lagishetty V., Katzka W., Rozengurt E., Jacobs J.P., Eibl G. (2019). Metformin alters the duodenal microbiome and decreases the incidence of pancreatic ductal adenocarcinoma promoted by diet-induced obesity. Am. J. Physiol. Gastrointest. Liver Physiol..

[67] Jeung W.H., Nam W., Kim H.J., Kim J.Y., Nam B., Jang S.S., Lee J.L., Sim J.H., Park S.D. (2019). Oral Administration of Lactobacillus curvatus HY7601 and Lactobacillus plantarum KY1032 with Cinnamomi Ramulus Extract Reduces Diet-Induced Obesity and Modulates Gut Microbiota. Prev. Nutr. Food Sci..

[68] Kubeck R., Bonet-Ripoll C., Hoffmann C., Walker A., Muller V.M., Schuppel V.L., Lagkouvardos I., Scholz B., Engel K.H., Daniel H. (2016). Dietary fat and gut microbiota interactions determine diet-induced obesity in mice. Mol. Metab..

[69] Shang Q., Shan X., Cai C., Hao J., Li G., Yu G. (2016). Dietary fucoidan modulates the gut microbiota in mice by increasing the abundance of Lactobacillus and Ruminococcaceae. Food Funct..

[70] Gao F., Lv Y.W., Long J., Chen J.M., He J.M., Ruan X.Z., Zhu H.B. (2019). Butyrate Improves the Metabolic Disorder and Gut Microbiome Dysbiosis in Mice Induced by a High-Fat Diet. Front. Pharmacol..

[71] Canfora E.E., Jocken J.W., Blaak E.E. (2015). Short-chain fatty acids in control of body weight and insulin sensitivity. Nat. Rev. Endocrinol..

[72] Sakamoto M., Takagaki A., Matsumoto K., Kato Y., Goto K., Benno Y. (2009). Butyricimonas synergistica gen. nov., sp. nov. and Butyricimonas virosa sp. nov., butyric acid-producing bacteria in the family ’Porphyromonadaceae’ isolated from rat faeces. Int. J. Syst. Evol. Microbiol..

[73] Shkoporov A.N., Khokhlova E.V., Chaplin A.V., Kafarskaia L.I., Nikolin A.A., Polyakov V.Y., Shcherbakova V.A., Chernaia Z.A., Efimov B.A. (2013). Coprobacter fastidiosus gen. nov., sp. nov., a novel member of the family Porphyromonadaceae isolated from infant faeces. Int. J. Syst. Evol. Microbiol..

[74] Jabari L., Gannoun H., Cayol J.L., Hedi A., Sakamoto M., Falsen E., Ohkuma M., Hamdi M., Fauque G., Ollivier B. (2012). Macellibacteroides fermentans gen. nov., sp. nov., a member of the family Porphyromonadaceae isolated from an upflow anaerobic filter treating abattoir wastewaters. Int. J. Syst. Evol. Microbiol..

