# Supplementary material for: Codium fragile Ameliorates High-Fat Diet-Induced Metabolism by Modulating the Gut Microbiota in Mice
Source: Nutrients. 2020 Jun 21;12(6):1848. doi: 10.3390/nu12061848 (PMC7353201; doi:10.3390/nu12061848)
Supplement: Supplementary file 1 [file nutrients-12-01848-s001.pdf]

Table S1. The compositions and formulas of chow diet and high-fat diet.

Class description	Ingredient (g/kg Diet)	Chow Diet		High-Fat Diet	
		g	Kcal	g	Kcal
Protein	Casein	200	800	200	800
Protein	L-Cystine	3	12	3	12
Carbohydrate	Corn Starch	397.49	1589.9	72.8	291.2
Carbohydrate	Dextrose	132	528	100	400
Carbohydrate	Sucrose	100	400	172.8	691.2
Fiber	Cellulose	50	0	50	0
Fat	Soybean Oil	70	630	25	225
Fat	Lard	0	0	177.5	1597.5
Mineral	Mineral mix	35	0	45	0
Vitamin	Vitamin mix	10	40	10	40
Vitamin	Choline Bitartrate	2.5	0	2	0
Anti-oxidant	t-BHQ	0.01	0	0	0
Total		1000	4000	858.1	4057
**Component**		**g (%)**	**Calorie (%)**	**g (%)**	**Calorie (%)**
Protein		20	20	24	20
Carbohydrate		63	63	40	34
Fat		7	16	24	45

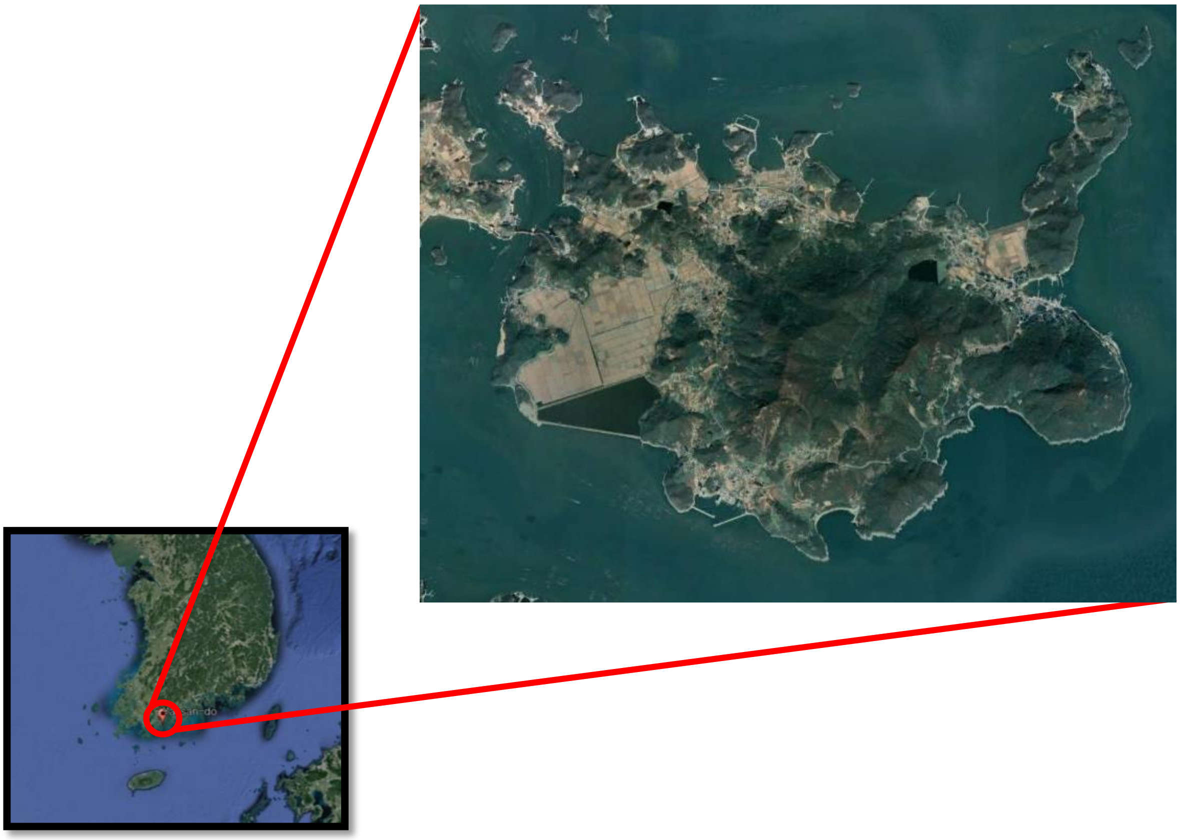

Figure S1. The *Codium fragile* sampling location (Yaksan-do; 34°22'41.23"N, 126°54'35.92"E). These maps were modified using Google Earth software.

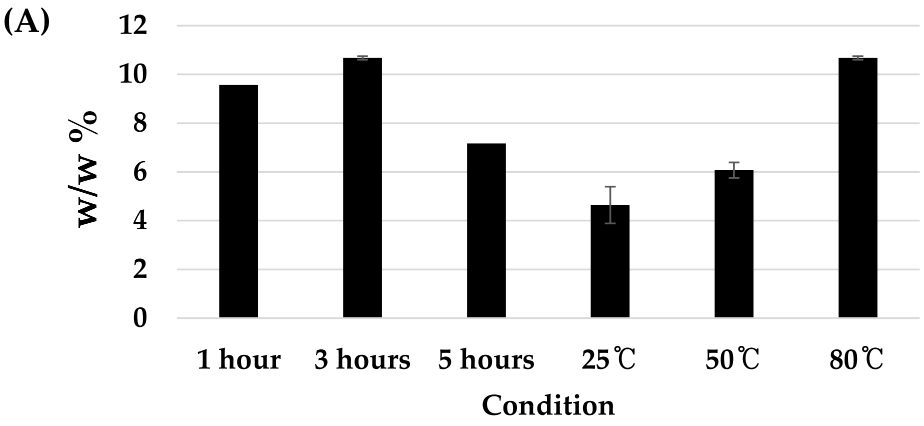

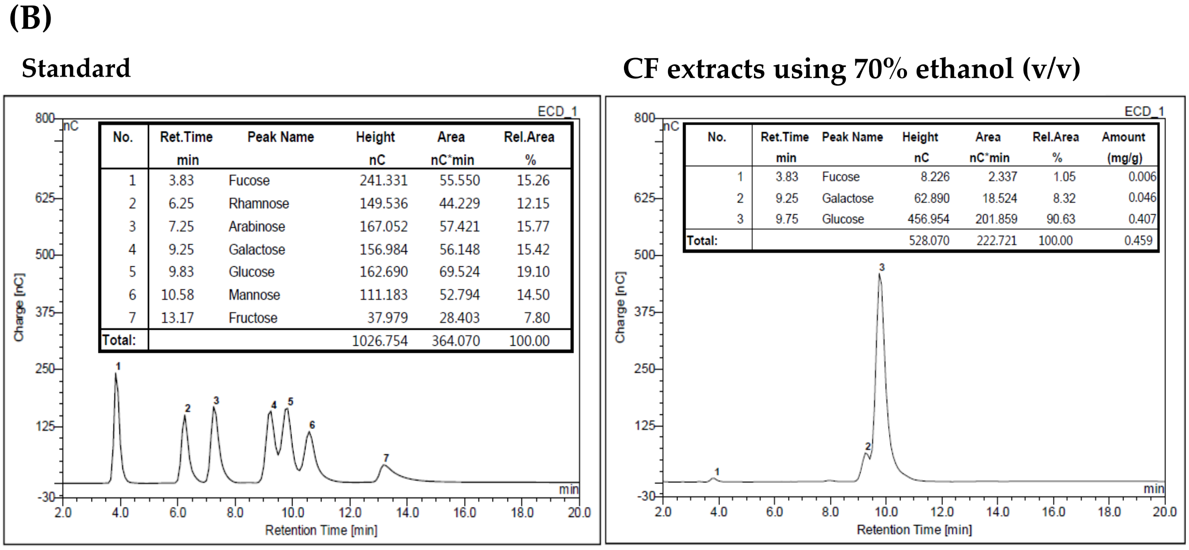

No.	Ret.Time min	Peak Name	Height nC	Area nC*min	Rel.Area %
1	3.83	Fucose	241.331	55.550	15.26
2	6.25	Rhamnose	149.536	44.229	12.15
3	7.25	Arabinose	167.052	57.421	15.77
4	9.25	Galactose	156.984	56.148	15.42
5	9.83	Glucose	162.690	69.524	19.10
6	10.58	Mannose	111.183	52.794	14.50
7	13.17	Fructose	37.979	28.403	7.80
Total:			1026.754	364.070	100.00

No.	Ret.Time min	Peak Name	Height nC	Area nC*min	Rel.Area %	Amount (mg/g)
1	3.83	Fucose	8.226	2.337	1.05	0.006
2	9.25	Galactose	62.890	18.524	8.32	0.046
3	9.75	Glucose	456.954	201.859	90.63	0.407
Total:			528.070	222.721	100.00	0.459

Figure S2. The extraction yield of CF extracts depending on both extraction time and extraction temperature (A) and the composition analysis of CF extracts (B).

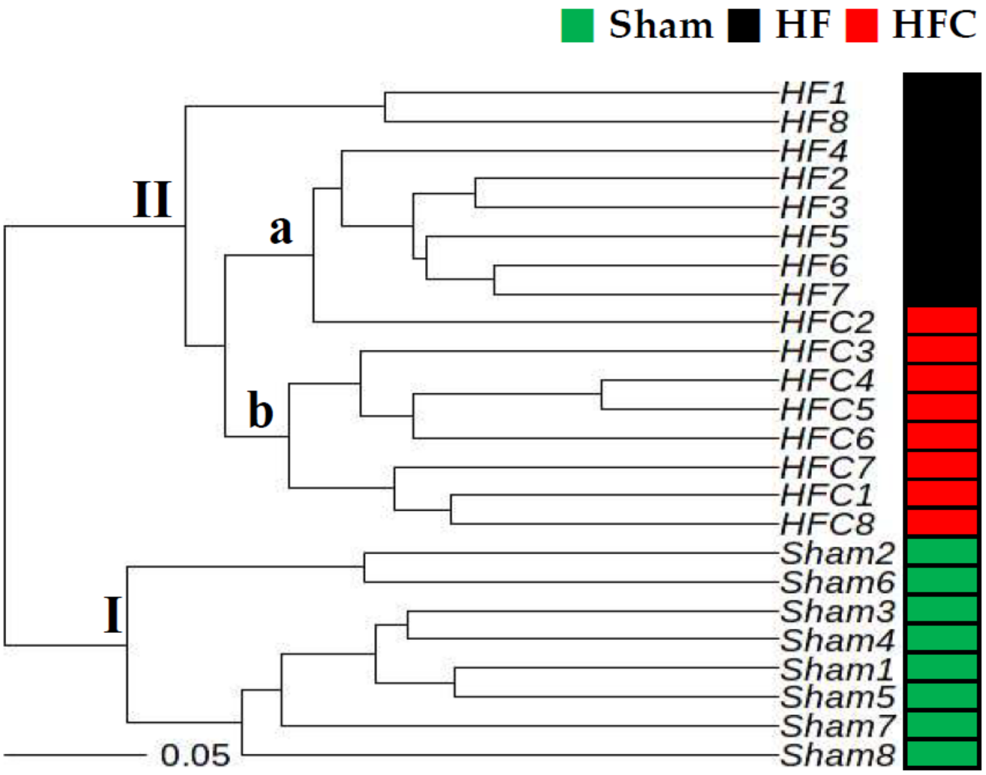

Figure S3. The clustering analysis of the gut microbiota using tree dendrogram based on braycurtis dissimilarity. Sham, HF and HFC indicated normal diet-fed group, high-fat diet-fed group, and high-fat diet plus CF extracts-fed group, respectively.
